# Artificial Intelligence Can’t Be Charmed: The Effects of Impartiality on Laypeople’s Algorithmic Preferences

**DOI:** 10.3389/fpsyg.2022.898027

**Published:** 2022-06-29

**Authors:** Marius C. Claudy, Karl Aquino, Maja Graso

**Affiliations:** ^1^College of Business, University College Dublin, Dublin, Ireland; ^2^Sauder School of Business, University of British Columbia, Vancouver, BC, Canada; ^3^Department of Management, University of Otago, Dunedin, New Zealand

**Keywords:** algorithm aversion, artificial intelligence, procedural justice, decision-making, impartiality

## Abstract

Over the coming years, AI could increasingly replace humans for making complex decisions because of the promise it holds for standardizing and debiasing decision-making procedures. Despite intense debates regarding algorithmic fairness, little research has examined how laypeople react when resource-allocation decisions are turned over to AI. We address this question by examining the role of perceived impartiality as a factor that can influence the acceptance of AI as a replacement for human decision-makers. We posit that laypeople attribute greater impartiality to AI than human decision-makers. Our investigation shows that people value impartiality in decision procedures that concern the allocation of scarce resources and that people perceive AI as more capable of impartiality than humans. Yet, paradoxically, laypeople prefer human decision-makers in allocation decisions. This preference reverses when potential human biases are made salient. The findings highlight the importance of impartiality in AI and thus hold implications for the design of policy measures.

## Introduction

Allocating scarce resources between groups and individuals is a perennial challenge of social life (Hardin, 1968). Deciding who is worthy of university admission, a loan to start a new business, or even an organ transplant involves trade-offs among competing claims and values. Decision-makers charged with the task of allocating such scarce resources face a daunting challenge. Because resources are finite, allocation decisions will benefit some and disadvantage others, and outcomes will often be perceived as unfair by some affected parties (Camps et al., 2019). Historically, nearly all such decisions were made by humans. What reliably emerges from both research and the common experience is that human decision-makers are not always impartial and often show systematic biases in judgment (Simon, 1951; Tversky and Kahneman, 1973, 1974; Gilovich et al., 2002). Studies show that desirable attributes of decision processes, like consistency, integrity, and impartiality (Thibaut and Walker, 1975; Leventhal, 1980), can be easily derailed by ingroup biases (Brewer, 1979; Hughes, 2017), biases against the outgroup (Hebl et al., 2020), or simple preference for those who offer instrumental value to the decision-maker (Cornelis et al., 2013; Zhao et al., 2015; Hughes, 2017). Regardless of the domain (e.g., HR or marketing), decisions are perceived as fairer when they are made without biases or prejudices, and when they are based on accurate information (Colquitt et al., 2013, 2018; Matta et al., 2017). Emphasizing the importance of using fair procedures to make decisions has long been advocated as one of the most effective ways to counteract the many biases that can undermine the acceptance and legitimacy of allocation decisions (Cropanzano et al., 2001, 2007; Miller, 2001; Helberger et al., 2020).

Recent advances in Artificial Intelligence (AI) computer systems that can sense, reason, and respond to their environment in real-time, often with human-like intelligence (Robert et al., 2020), have made many optimistic that AI will soon eliminate human biases and overcome the limitations that often lead to injustice and suboptimal allocation decisions (Ghahramani, 2015; Silver et al., 2017; Singh et al., 2017; Mozer et al., 2019; Glikson and Woolley, 2020). Indeed, forecasts predict that decision-makers will increasingly turn to AI when allocating scarce resources in domains such as business, law, and healthcare (Brynjolfsson and Mitchell, 2017; Fountaine et al., 2019; Frank et al., 2019; Rawson et al., 2019). The use of algorithms that rely on big data holds the promise of debiasing decision-making procedures by removing human subjectivity typically inherent in judging and comparing individuals (Newman et al., 2020). For example, much work has focused on utilizing AI to detect bribery and other forms of corruption to eliminate impartiality violations in governmental and other organizational contexts (Köbis and Mossink, 2021).

Despite their apparent advantages as decision-making tools, people’s trust in AI often lags behind its rising capabilities, and many are averse to turning over allocation decisions to non-human entities (Glikson and Woolley, 2020). Their aversion has been traced to a belief that machines do not possess a complete mind and, therefore, cannot freely choose actions, nor can they adequately reflect on their consequences (Johnson, 2015; Bigman and Gray, 2018; Bigman et al., 2019). Furthermore, Newman et al. (2020) found that people perceive algorithmically-driven decisions as less fair because of AI’s inability to consider and contextualize qualitative information. Castelo et al. (2019) also investigated people’s aversion to relying on algorithms to perform tasks previously done by humans and found that algorithms are trusted and relied on more for tasks that require cognitive abilities and rationality vs. tasks that depend more on emotional intelligence or intuition.

However, whether laypeople perceive AI as more impartial than human deciders has not been explicitly addressed. The aim of the present study is to bridge research on procedural justice (Leventhal, 1980) with algorithm aversion to explain how laypeople’s impartiality perceptions between AI and human decision-makers differ (Study 1), and whether impartiality violations shift people’s preferences for AI in allocation decisions (Study 2 and 3).

## Procedural Justice, Impartiality, and Algorithmic Preferences

Philosophers and scholars have offered several perspectives on why just procedures matter (Rawls, 1971/1999; Leventhal, 1976, 1980; Lind and Lissak, 1985; Tyler et al., 1985; Sheppard and Lewicki, 1987; Tyler, 1988; Sheppard et al., 1992). In organizational and legal contexts, procedural justice is concerned with people’s fairness perceptions regarding the processes or rules applied throughout the decision-making process (Thibaut and Walker, 1975). When it comes to the allocation of scarce resources like getting a job or securing a loan, procedures will be seen as fairer when they are impartial, i.e., if procedures are applied consistently and without biases; are based on accurate information; are correctable and ethical, and are representative of relevant parties involved in the decision (Leventhal, 1980). Impartiality means that when people are making moral decisions (e.g., allocating scarce goods and resources) they should not give any special treatment to themselves, or to members of their own ingroup, and instead take a neutral and unbiased position (Cottingham, 1983).

Decades of organizational justice research show that impartiality perceptions are positively associated with cooperation, performance, or job satisfaction whilst reducing potentially damaging behaviors and attitudes such as retaliation, complaints, or negative word-of-mouth (Cohen-Charash and Spector, 2001; Colquitt et al., 2013; Colquitt and Zipay, 2015). Furthermore, knowing that procedures are impartial can make people more accepting of authorities, laws, and policies, even when the outcomes are disadvantageous to them (Tyler, 1994; Sunshine and Tyler, 2003).

One of the greatest hopes regarding algorithmically-driven decisions in organizational contexts lies in AI’s ability to suppress or even eliminate common human biases that threaten the enactment of fair procedures (Graso et al., 2019). AI has the potential for standardizing decision-making processes, thereby eliminating many of the idiosyncrasies that can lead human decision-makers to depart from impartiality (Grace et al., 2018; Raisch and Krakowski, 2020). Giubilini and Savulescu (2018), for example, argue that AI could serve as an “artificial moral advisor” because of its ability “to take into account the human agent’s own principles and values” whilst making consistent judgments without human biases and prejudice. In principle, using AI in allocation decisions should thus increase impartiality by “standardizing decision procedures and reducing potential conflicts through highly consistent and supposedly unbiased decisions” (Ötting and Maier, 2018), which directly correspond to tenets of fair procedures (Leventhal, 1980).

However, empirical evidence regarding laypeople’s perceptions of impartiality in algorithmically-driven decisions is limited, and findings in adjacent domains are ambiguous (for an overview, see Castelo et al., 2019; Glikson and Woolley, 2020). For example, Newman and colleagues find that algorithm-driven (vs. human-driven) hiring and lay-off decisions are perceived as less fair because people view algorithms as reductionist and unable to contextualize information (Newman et al., 2020). Yet, the authors do not test for perceptional differences between human and AI deciders. Furthermore, Ötting and Maier (2018) found that procedural justice had a positive impact on employee behaviors and attitudes, irrespective of whether the decider was a human, a robot, or a computer system. One limitation of their study was that the authors manipulated procedural justice (fair vs. unfair) and did not explicitly measure people’s baseline perceptions regarding human vs. AI deciders. Other evidence suggests that people might associate greater impartiality with AI-based decision procedures. For example, people generally perceive robots and artificial intelligence systems as consistent and reliable (Dzindolet et al., 2003), and they are more likely to rely on algorithmic advice than on human advice, particularly when their expertise is limited (Logg et al., 2019). In comparison, there is ample evidence to suggest that human decisions are often biased, for example, by prejudice (Hebl et al., 2020) or favoritism toward people who are close to them (Hughes, 2017). It is, therefore, reasonable to posit that people are more likely to attribute greater impartiality to AI than to a human decision-maker because they will view the former as having more of the attributes that characterize an unbiased decision-maker. Formally, we thus argue that

*H1* = *Laypeople will associate greater impartiality with AI (vs. human) decision-makers in allocation decisions*.

We are not assuming that people will believe that AI is entirely impartial, only that they will be viewed as *more* capable of approaching this standard than humans. If so, based on considerable evidence suggesting that people value impartiality and bias-suppression in decision-makers (Leventhal, 1980), people should prefer an AI over human-decision makers in contexts in which impartiality by human deciders is potentially jeopardized. That is because implementing AI holds the potential to remove subjective (and potentially biased) judgments from allocation decisions and instead make those decisions on more objective or quantifiable competence criteria. It seems possible, therefore, that laypeople who may be subjected to biased evaluations, especially when these judgments are based on prejudice or stereotypes, should prefer AI decision-makers over humans. Formally, we posit that

*H2* = *When standards of impartiality are potentially violated, laypeople show greater preferences for AI (vs. human) decision-makers in allocation decisions*.

## Materials and Methods: All Studies

In an exploratory study, we first test whether people perceive allocation decisions that involve AI as more impartial compared to procedures that are led by humans (Study 1). Next, we test whether laypeople’s preference for AI (vs. human) decision-makers shifts if they are prompted to think that a human decision-maker might be partial (Study 2 and 3). Our studies comply with ethical regulations regarding human research participants, and we received ethical approval from the Human Research Ethics Committee at a major European university. We obtained informed consent from all participants. We informed them that participation was voluntary and that they could stop their participation at any time. We recruited all participants from Prolific Academic (Peer et al., 2017). All our studies contain measures of gender and age. Demographic information and sample sizes for each study are presented in Table 1. Except for Study 1, all studies were pre-registered.

**TABLE 1:** Samples’ demographic information.

	*N* recruited	*N* retained*	% Male	Age *M*	Age *SD*
Study 1	120	118	58.3	31.6	13.5
Study 2	440	369	51.1	32.7	11.5
Study 3	323	318	48.4	34.6	12.2

*We eliminated responses from participants who failed attention or crucial comprehension check questions. We specify our elimination strategy for each study in the text.

All measures, manipulations, and exclusions are disclosed in the “Materials and Methods” section and Supplementary Material. We determined sample sizes by assuming a medium effect (Cohen’s *d* = 0.50), and we conducted a power analysis to calculate the required number of participants per condition to obtain a power of 0.95. Data collection was stopped once the pre-determined sample size was reached. All studies included attention or comprehension checks, which resulted in the exclusion of participants who failed those checks. The complete stimulus material and data can be publicly accessed in Supplementary Material. Test statistics presented in this research are all two-sided.

## Study 1

### Materials and Methods

In this exploratory study, we tested our hypothesis that laypeople perceive AI and human decision-makers differently regarding impartiality. We informed participants that they would be presented with two decision procedures and that they had to indicate which of these they would prefer if the decision were being made about them. Participants were asked to assume that they had applied for positions at two different companies. They then learned that “both companies consider your qualifications, experience, and skills before making a decision” and that “the process also involves assessing the likelihood of you performing well on the job and your fit with the company culture.” The description of both companies read as follows:

***Company A*** *uses a skilled and experienced Human Resource (HR) manager to evaluate your application and to assess your suitability for the position. In this process, the HR manager uses his/her personal judgment to decide whether you should be hired.*

***Company B*** *uses a highly sophisticated computer program that relies on Artificial Intelligence (AI) to automatically evaluate your application and to predict your suitability for the position. In this process, the computer program uses large amounts of data to decide whether you should be hired.*

The order in which Company A and B were presented was randomized.

#### Measures

##### Impartiality Perceptions

In this exploratory study, we asked participants to compare the two decision procedures on procedural justice dimensions (Thibaut and Walker, 1975; Leventhal, 1980), including impartiality. This exploratory study included several items that are not relevant in the context of this study, but are nevertheless included in Supplementary Material. We asked participants to use a slider scale to indicate whether they believed that the decision procedure involving the human (−10) or the AI (+ 10) would be more impartial. Additionally, we asked participants to indicate which procedure would give them a better chance of getting the job.

##### Choice

We asked participants to indicate which one of these decision procedures they would prefer if the decision was being made about them (1 = *HR manager*; 2 = *AI*).

##### Open-Ended Rationale

We also asked participants to briefly explain their choice using a minimum of 100 characters. We used these answers to further inform our theorizing regarding the role of impartiality in allocation decisions.

### Results

Supporting our prediction, results of the single-sample *t*-test showed that people perceive AI as more impartial than humans, [*t*(118) = 8.82; *p* < 0.001; *M* = 4.18, *SD* = 5.20, 95% CI = (3.31–5.12]; Cohen’s *d* = 0.812]. Furthermore, a χ^2^-test revealed that the majority of people (76.7%) preferred human deciders over AI if the decision was being made about them, χ^2^(1) = 34.13, *p* < 0.001. Notably, this preference emerged even though people perceived the AI as more impartial than a human. Finally, results showed that people associated a higher chances of getting the job with the human decider [*t*(118) = –6.36; *p* < 0.001; *M* = –2.68, *SD* = 4.60, 95% CI = (–3.52 to –1.85); Cohen’s *d* = 0.58].

#### Qualitative Responses

The qualitative answers revealed that a significant proportion of people who chose the AI decider (93%) did so because they felt it was less biased and more impartial than a human decision-maker. For example, one respondent wrote: *“I think humans are inherently biased*… *I would prefer to be judged solely on my skills and experiences and I think a computer program would do a better job of this because it would not be swayed by my gender or appearance.”* In comparison, explanations for the choice of human deciders often involved opposite justifications. As one participant wrote: *“I think I would react better toward a person than a machine. The machine doesn’t take into account my charm,”* hinting that they would have a better chance at influencing a human than a machine.

#### Discussion

Our results support previous research on algorithm aversion by showing that most people prefer humans over AI to make allocation decisions. At the same time, participants believe that an AI would be more impartial than human decision-makers, thus supporting hypothesis 1.

## Study 2

Study 1 showed that people perceive AI (vs. human) decision-makers to be more impartial. Based on research we reviewed that shows that people value impartial and unbiased decision-makers in allocation decisions, laypeople’s preferences for AI decision-makers should thus shift when they believe that a human decision-maker might be partial. We test this possibility in Study 2 by examining people’s preference for an AI over a human when the human is likely to be biased in favor of them, against them, or when they know the human is biased but are uncertain of the direction. We expected people to prefer AI (vs. human) decision-makers when they know that a person is negatively (vs. positively) biased against them.

### Materials and Methods

Assuming a medium effect size (Cohen’s *d* = 0.50), power analysis suggested that we need 104 participants per condition to obtain a power of 0.95. To allow for participants failing the attention check, we aimed for 130 participants per condition. The study was pre-registered at: https://aspredicted.org/blind.php?x=xi9es2. Sample characteristics are provided in Table 1.

We utilized a mixed design in which a single factor was varied between subjects: a human decision-maker that was *partial in their favor* (i.e., the decider prefers people who have certain characteristics which the participant possesses); *partial in a way that disadvantaged them* (i.e., decider prefers people who have certain characteristics which the participant does not possess); or partial in an *uncertain* way that could favor or disadvantage them (i.e., the decider is partial, but it is unclear whether they are partial toward the participant).

As a within-subject factor, all participants responded to four scenarios presented in random order in which they had to specify whether they preferred a partial human or an impartial AI to make hiring, lay-off, university admission, and loan approval decisions. For example, in the university admission scenario, participants were informed that “a university is deciding which students to admit to their incoming class.” Participants then learned that the decision was either made by:

A. An admissions officer who has preferences to admit the best students who also fit certain demographic categories such as race, gender, age or social class. It turns out that this admissions officer has preferences for characteristics (*you possess/you do not possess/don’t know if you possess or not*), which makes it likely that the officer will be (*favorable/unfavorable/either favorable or unfavorable*) toward you when evaluating your application.

Or;

B. A sophisticated computer program that has been programmed to automatically identify and admit the best students, irrespective of demographic factors such as nationality, gender, age, or social class. The program shows no preferences for demographic characteristics, which means that the program will evaluate your application solely on your academic qualifications. All admissions decisions will be made by the program, with no human input.

The full description of our vignettes can be found in Supplementary Material.

#### Measure

##### Choice

The dependent variable in this study was a dichotomous choice, where we asked people: “Which decision procedure would you prefer if you wanted to secure this (outcome: get this job; obtain this loan; be admitted to this University course; keep your job)?” (human vs. AI).

### Results

**FIGURE 1:**
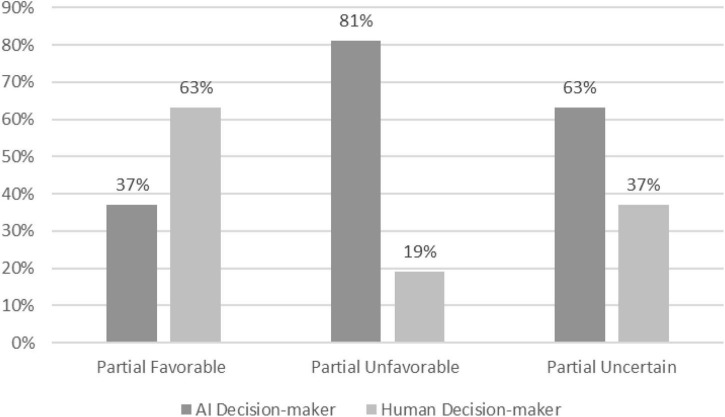
Preferences for AI decision-makers in favorably biased, unfavorably biased and uncertain conditions (Study 2). χ^2^(2), (*N* = 369) = 60.88; *p* < 0.001.

Our pre-registered plan was to analyze this between-subject study and test our second hypothesis using a chi-square difference test. Across the four decisions scenarios, a Chi-squared test, χ^2^(2), (*N* = 369) = 60.88; *p* < 0.001, revealed that people were more likely to choose a human (63%) when the decision-maker was partial in their favor and less likely to select a human (19%) when the decider was biased against them (Figure 1). A majority chose the AI (63%) when they did not know whether the human decider would be biased favorably or unfavorably toward them (uncertain condition).

To test the robustness of these findings and detect differences between the four decision contexts, we also conducted an additional (i.e., not pre-registered) repeated measures ANOVA where we treated the preference for AI in each scenario as a repeated measure. Although the dependent variable is binary, prior research has suggested that such violations of normality might be largely inconsequential (Glass et al., 1972). The ANOVA confirmed a significant main effect of condition on choice, [*F*(2, 366) = 50.38, *p* < 0.001, η^2^partial = 0.22]. This finding was further supported by *post hoc* tests, which showed that choices in all conditions were significantly different. We found a small but significant main effect for the scenario, [*F*(3, 366) = 12.78, *p* < 0.001, η^2^partial = 0.03], but not for scenario × condition interaction, [*F*(6, 366) = 0.86, *p* = 0.523, η^2^ partial = 0.01], providing further support that the results are robust across multiple decision contexts.

### Discussion

The results provide evidence that impartiality constitutes an essential determinant of laypeople’s preferences for AI in allocation decisions. Specifically, people showed greater preferences for an AI when they believed that human decision-makers might show prejudice or negative biases toward them, thus supporting hypothesis 2. Only if the human was partial in their favor did people prefer the human decision-maker. The finding thus highlights an important boundary condition to people’s algorithm aversion, which has been observed across a broad range of decision contexts (Bigman and Gray, 2018; Young and Monroe, 2019; Glikson and Woolley, 2020).

## Study 3

In our final study, we examined whether people’s preferences for an AI (vs. human) might reverse when they believe that a human decision-maker might be partial against them because of their social status within their profession. We selected respect as a form of one’s social resource (Foa and Foa, 1980; Brown et al., 2020) which may make a human more partial than AI. The study thus aimed to replicate and advance findings from Study 2.

### Materials and Methods

In this study, we utilized a different manipulation of partiality. Specifically, we varied the degree to which people were respected by others within their profession based on their status. Assuming a medium effect size (Cohen’s *d* = 0.50) and a statistical power level of 0.85, power analysis suggests that we needed a minimum of 142 participants per condition to obtain a power of 0.95 for a two-tailed hypothesis test. We recruited 320 participants (160 per condition) to allow for failed attention checks. The study was pre-registered at: https://aspredicted.org/aw8ax.pdf. The full sample characteristics are shown in Table 1.

### Procedures

We utilized a between-subject design in which we varied partiality (favorable vs. unfavorable) between subjects. People were told that they were highly respected because of their status (partial in their favor), or looked down upon (partial against them) by others within their profession (Brown et al., 2020). Specifically, we asked people to imagine a situation that could occur in everyday work life. The scenario read as follows:

“You are a retail manager. You’ve been working at a large (*small*) and very prestigious (*insignificant*) retail store in the US for most of your career and generally enjoy what you do. Your role is (*not*) very well respected in your profession and generally, people in your industry highly respect (*look down upon*) you and your work. As a result, many (*very few*) people in your industry have supported you in your career progression.

Last week you applied for a new managerial position at a large department store, where you and other applicants will have to perform an online job interview. Overall, when you interview for new roles, you are very highly respected (*looked down upon*).”

We then asked participants to write down three potential upsides (downsides) of having their current job, when applying for a new position.

Next, participants learned that the manager of the retail store “informed them that they can choose to be interviewed by the current manager or a highly sophisticated Artificial Intelligence (AI) software.” We then provided participants with an explanation of the interview processes, informing them that “during the human-led (AI-led) interview, the current manager (a highly sophisticated AI) will ask you a series of questions about your previous work experience and will use your answers to assess your suitability for the position. In this process, the manager (AI) will use personal judgment and experience (an advanced algorithm) to determine whether you are the right fit for the position. The final hiring decisions will be made by the manager (AI).”

#### Measures

##### Choice

The dependent variable in this study was a dichotomous choice. We asked participants: “Please select who you prefer to interview you for the position at the department store (manager vs. AI).”

##### Procedural Justice

Furthermore, we included the impartiality items from Studies 1 and 2 and asked people to indicate “how important were the following criteria for you in choosing your interviewer?,” and measured their responses on a scale from 1 (*not at all important*) to 5 (*extremely important*).

##### Manipulation Check

To test whether our manipulation induced a sense of favorable (vs. unfavorable) status evaluations, we asked participants to complete a three-item scale (α = 0.87): “I feel that the other job candidates are more qualified than I am”; “I feel that the other candidates have more status than I do”; “I feel that the other job candidates are more experienced than I am.” They noted their responses on a scale from 1 (*strongly disagree*) to 5 (*strongly agree*).

##### Realism Check

We assessed realism with two items (α = 0.75). Participants indicated the extent to which they agree that “the presented scenario was realistic” and whether they could “imagine being in the described situation.” They noted their responses on a scale from 1 (*strongly disagree*) to 7 (*strongly agree*).

### Results

The manipulation check shows that people in the unfavorable partiality condition (*M* = 3.20, *SD* = 1.03) felt less qualified than in the favorable condition (*M* = 2.20, *SD* = *0.89*), [*t*(316) = –9.37, *p* < 0.001, *d* = 1.05]. Furthermore, participants felt that the presented scenario was realistic (*M* = 5.27, *SD* = 1.23).

**FIGURE 2:**
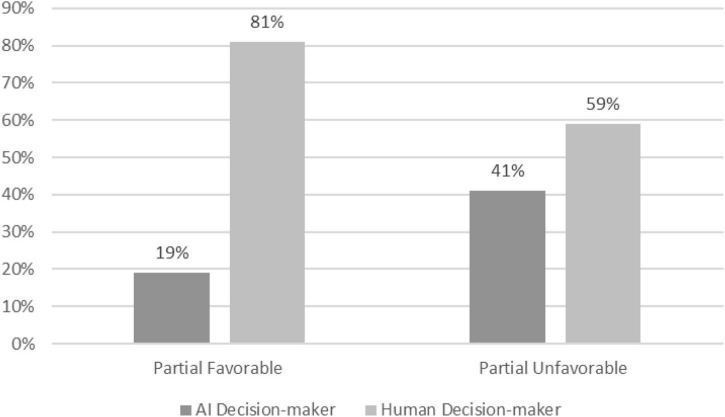
Preferences for AI decision-makers in favorably biased vs. unfavorably biased conditions (Study 3). χ^2^(1), (*N* = 318) = 18.62; *p* < 0.001.

Our pre-registered plan was to analyze this between-subject study and test our second hypothesis using a chi-square difference test. Across the two partiality conditions, a Chi-squared test, χ^2^(1), (*N* = 318) = 18.62; *p* < 0.001, revealed that only 19.3% of people wanted to be interviewed by the AI when they believed that others were partial in their favor. In comparison, 41.4% of people who thought that others might be partial against them preferred to be interviewed by an AI (Figure 2). The results thus lend further support to our hypothesis that algorithmic preferences are conditional upon people’s perception of the impartiality of human decision-makers.

An independent samples *t*-test showed that participants in the unfavorable condition placed greater importance on bias suppression (*M* = 4.14, *SD* = 1.088) and impartiality, (*M* = 4.03, *SD* = 1.019) compared to people in the favorable condition (*M* = 3.84, *SD* = 1.175; *M* = 3.74, *SD* = 1.128), [*t*(316) = –2.366, *p* < 0.019, *d* = 0.265]; [*t*(316) = –2.414, *p* < 0.016, *d* = 0.271], respectively. No other differences were detected.

### Discussion

Study 3 offered further evidence in support of hypothesis 2, i.e., people’s preferences for algorithmically-driven allocation decisions depend on the impartiality of the decision-maker. In the unfavorable condition, more than twice as many people chose to be interviewed by an AI compared to the favorable group. The results suggest that these differences can be explained by the greater importance people place on bias suppression and impartiality when they are subjected to unfavorable evaluations by others.

## General Discussion

Technological advances are expected to result in increased use of AI in decisions that distribute scarce goods and resources between competing parties. The transition toward AI-led decision-making raises important moral and ethical questions for businesses, many of which concern algorithmic fairness and transparency (Rahwan et al., 2019; Raisch and Krakowski, 2020). But despite the critical importance of these issues, we still have a limited understanding of how people perceive allocation decisions in which human deciders are replaced by artificial forms of intelligence. While a growing body of work has explored the cognitive-affective factors behind people’s algorithm aversion (Khalil, 1993; Glikson and Woolley, 2020; Raisch and Krakowski, 2020; Robert et al., 2020), few studies have investigated the role of impartiality in human-AI interactions (Leventhal, 1980). Our study addresses this paucity and contributes to the literature by highlighting an important boundary condition to laypeople’s algorithm aversion.

First, our study sheds new light on the role of impartiality in human-AI interactions. Since AI is not freighted with some of the characteristics that can lead humans to stray from impartiality, it holds the promise of enhancing the accuracy, consistency, and incorruptibility from the social influence of many decision procedures. Importantly, impartiality has been shown to influence the acceptance and legitimacy of decision procedures (Tyler et al., 1985). People consistently value impartiality and prefer deciders who make allocation decisions without biases, prejudices, or previously determined personal preferences (Miller, 2001; Shaw and Olson, 2014). While we provide further evidence that people prefer humans over AI in decisions concerning them (Study 1), we consistently show that laypeople associate greater impartiality with AI (vs. human) decision-makers. This finding is noteworthy because technologists, scholars, and policy-makers have often raised concerns regarding the prevalence of partiality in AI that stems from historically biased data and poorly designed algorithms (Khalil, 1993; De Cremer, 2020; Robert et al., 2020). Our findings suggest that laypeople perceive AI as more capable of achieving impartiality than humans (Helberger et al., 2020). This is not to say that laypeople believe that AI is completely unbiased—it merely suggests that despite the many flaws and limitations within algorithmic decision making, laypeople still perceive AI to be less biased than humans.

Secondly, our findings show that impartiality concerns constitute an important boundary condition to people’s algorithmic preferences (Study 2 and 3). We show that when laypeople are concerned about the negative biases of human deciders, their preferences shift toward AI decision-makers. This is because they emphasize impartiality and bias suppression, which AI is perceived to be more capable of than human decision-makers. In other words, people who are potentially subjected to negative biases show greater preferences for AI deciders because it increases impartiality and removes biases, which might curb their chances of obtaining desired outcomes (e.g., securing a job or getting admitted to a university). The only exception is when human decision-makers are partial in people’s favor, in which case most people prefer the human over an AI. For a person who potentially benefits from a partial decider, choosing an AI to make decisions about them might even be self-destructive. This finding also has important implications for AI ethics. While previous studies have suggested that designing policies and regulations that continue to build trust in AI is likely to enhance further the acceptance and legitimacy of AI-led decisions (Khalil, 1993; Glikson and Woolley, 2020), we have identified an important caveat to this goal. Namely, despite its positive features and potential for standardizing and debiasing decision procedures (Zou and Schiebinger, 2018), people might not actually wish to endorse AI if they believe that partial decision-makers will help them to attain desired outcomes. Therefore, AI’s capabilities are likely to be valued more by those who experience negative evaluations or even prejudice in intra-human interactions.

In our studies, we only assessed prejudice (Study 2) and respect based on one’s status (Study 3) as examples of partiality that may lead people to endorse AI decision-makers. Future research could examine whether other impartiality violations might lead people to support AI against human-human interactions. Future research could also investigate the moderating role of related constructs like power (Fast and Schroeder, 2020). For example, in some instances, an AI might be perceived as less of a threat to one’s position in the organizational hierarchy. Indeed, research has shown that people prefer to be replaced by robots (vs. humans) when their job loss is at stake (Granulo et al., 2019).

Our study also offers managerial implications. Despite significant value being placed on justice, fair procedures are still frequently ignored, and decision-makers routinely deviate from principles of impartiality that they often claim to value (Graso et al., 2019). When this is the case, implementing AI-led decision procedures might provide some way to improve the accuracy, consistency, and impartiality of organizational decision procedures. However, such changes might also be met by resistance from employees and other stakeholders who have little to gain from such changes. While people who are concerned with being evaluated negatively are more likely to endorse such changes, people who benefit from partiality in the organizations are more likely to resist handing over decisions to non-human entities. Indeed, our study suggests (and future research can attest) how social resource-rich groups may be less invested in endorsing impartial decision-making tools such as AI. Future research should further explore how resistance to AI decision-makers, specifically among powerful individuals, can be overcome via algorithmic design or supporting procedures and policies.

In summary, our set of pre-registered studies conducted across diverse and complementary contexts has notable strengths. It builds on existing findings and advances our understanding of people’s perceptions of AI vs. human decision-makers. Furthermore, we have identified perceived impartiality as an important boundary condition to people’s algorithmic preferences in allocation decisions. Our simple design involved repeatedly contrasting the two decision-makers, and it allowed us to identify people’s underlying and reflexive assumptions regarding the impartiality of AI as decision-makers, and how they influence preferences for AI in different contexts. Nonetheless, our study has caveats and limitations, which we discuss with the hope of encouraging future research.

## Limitations and Opportunities for Future Research

First, we only assessed people’s perceptions of AI systems that do not influence participants directly. Throughout our studies, we asked participants to assume that these decisions affect them. Still, as our participants were online platform users, there were no real consequences to their strongly endorsing AI or human decision-makers. Scarce research resources permitting, we recommend that future studies assess whether impartiality influences people’s preference for human or AI decision-makers when they themselves are invested in the outcome in question. An example would be giving employees in a large company an option to choose an AI or a human decision-maker when assessing promotions, raises, or bonuses. Furthermore, emerging research shows that in real-life scenarios, people might fail to reliably detect differences between algorithmically-generated and human-generated content, and that stated preferences might therefore diverge from actual behavior (Köbis and Mossink, 2021).

Second, while this is not explicitly tested in this study, it is possible that the use of AI in allocation decisions would have stronger support among people who believe that they might be disadvantaged or who experience prejudice because of their race, gender, or sexual orientation (Zou and Schiebinger, 2018). On the other hand, this support might be tempered by concerns that a more impartial decision-maker might not produce the outcomes they desire. We leave it to future research to investigate this possibility.

Finally, we focused on people’s perceptions of the human vs. AI decision-makers, but we did not examine attitudes toward the calibrators behind the AI. Any algorithm-based system is only as good as its inputs, and those inputs are only as good as the person calibrating the system. Perhaps people’s trust in AI as an impartial entity is inextricably linked with their trust in the AI’s calibrator. The appeal of certain AI systems is that they are self-correcting and capable of learning (Silver and Silver, 2017; Silver et al., 2017) which should presumably increase people’s perceptions of AI as distinct entities with minds of their own (Bigman and Gray, 2018; Bigman et al., 2019). Alternatively, we may witness a reality in which AI will remain simple reflections of their human masters and their calibrating powers, incapable of ever achieving true impartiality.

## Data Availability Statement

The original contributions presented in this study are included in the article/Supplementary Material, further inquiries can be directed to the corresponding author/s.

## Ethics Statement

The studies involving human participants were reviewed and approved by the UCD Office of Research Ethics, University College Dublin, Belfield, Dublin (No. HS-E-19-102-CLAUDY). The patients/participants provided their written informed consent to participate in this study.

## Author Contributions

MC co-designed and ran the studies, wrote up the results, and wrote parts of introduction and discussion. KA provided overall guidance regarding the direction of the study, co-designed studies, and wrote parts of introduction and discussion. MG provided input on theoretical framing and study design and wrote parts of introduction and discussion. All authors contributed to the article and approved the submitted version.

## Conflict of Interest

The authors declare that the research was conducted in the absence of any commercial or financial relationships that could be construed as a potential conflict of interest.

## Publisher’s Note

All claims expressed in this article are solely those of the authors and do not necessarily represent those of their affiliated organizations, or those of the publisher, the editors and the reviewers. Any product that may be evaluated in this article, or claim that may be made by its manufacturer, is not guaranteed or endorsed by the publisher.
