# Supplementary material for: Artificial Intelligence Can’t Be Charmed: The Effects of Impartiality on Laypeople’s Algorithmic Preferences
Source: Front Psychol. 2022 Jun 29;13:898027. doi: 10.3389/fpsyg.2022.898027 (PMC9277554; doi:10.3389/fpsyg.2022.898027)
Supplement: Supplementary file 1 [file Data_Sheet_1.docx]

**Supplementary Material**

**STUDY 1**

**Imagine you apply for positions at two different companies.**

Both companies consider your qualifications, experience, and skills before making a decision. The process also involves assessing the likelihood of you performing well on the job and your fit with the company culture.

**Company A** uses a skilled and experienced Human Resource (HR) manager to evaluate your application and to assess your suitability for the position. In this process, the HR manager uses his/her personal judgement to decide whether you should be hired**.**

**Company B** uses a highly sophisticated computer program that relies on Artificial Intelligence (AI) to automatically evaluate your application and to predict your suitability for the position. In this process, the computer program uses large amounts of data to decide whether you should be hired.

S**TUDY 2**

**A university is deciding which students to admit to their incoming class. The decision will be made in either of the following ways:**

A. By an admissions officer who has preferences to admit the best students who also fit certain demographic categories such as race, gender, age or social class. It turns out that this admissions officer has preferences for characteristics you possess, which makes it likely that the officer will be favorable towards you when evaluating your application.

B.  By a sophisticated computer program that has been programmed to automatically identify and admit the best students, irrespective of demographic factors such as nationality, gender, age, or social class. The program shows no preferences for demographic characteristics, which means that the program will evaluate your application solely on your academic qualifications. All admissions decisions will be made by the program, with no human input.

**A bank is deciding whether to give personal loans to applicants. The decision will be made in either of the following ways:**

A. By a loan officer who has preferences to approve loans of people who are financially eligible and also fit certain demographic categories such as race, gender, age or social class. It turns out that this loan officer’s preferences are for characteristics you possess, which makes it likely that the loan officer will be favorable**towards you**when evaluating your loan application.

B. By a sophisticated computer program that has been programmed to automatically evaluate and approve loans for people who are financially eligible, irrespective of demographic factors such as nationality, gender age, or social class. The program shows no preferences for demographic characteristics, which means that the program will evaluate your loan application solely on your financial eligibility. All loan decisions will be made by the program, with no human input.

**A company is deciding who to hire for an open position. The decision will be made in either of the following ways:**

A. By a supervisor who has preferences to hire the most qualified candidates, who also fit certain demographic categories such as race, gender, age or social class. It turns out that this supervisor's preferences are for characteristics you possess, which makes it likely that the supervisor will be favorable towards you when evaluating your application.

B.  By a sophisticated computer program that has been programmed to automatically evaluate and identify the most qualified candidates, irrespective of demographic factors such as nationality, gender age, or social class. The program shows no preferences for demographic characteristics, which means that the program will evaluate your application solely based on your vocational qualifications. All hiring decisions will be made by the program, with no human input.

**A company is deciding which employees to layoff for a downsizing initiative that is required to keep the company in business. The decision will be made in either of the following ways:**

A. By an HR manager who has preferences to retain the most productive people who also fit certain demographic categories such as race, gender, age or social class. It turns out that this manager's preferences are for characteristics you possess, which makes it likely that the manager will be favorable towards you when evaluating whether you should be laid off or not.

B.  By a sophisticated computer program that has been programmed to automatically identify and keep the most productive employees, irrespective of demographic factors such as nationality, gender age, or social class. The program shows no preferences for demographic characteristics, which means that the program will evaluate whether you should be laid off or not solely based on your productivity. All layoff decisions will be made by the program, with no human input

**Unfavorable Condition**

**A university is deciding which students to admit to their incoming class. The decision will be made in either of the following ways:**

A. By an admissions officer who has preferences to admit the best students who also fit certain demographic factors such as race, gender, age or social class. It turns out that this admissions officer has preferences for characteristics you do not possess, which makes it likely that the officer will be unfavorable towards you when evaluating your application.

B.  By a sophisticated computer program that has been programmed to automatically identify and admit the best students, irrespective of demographic factors such as nationality, gender, age, or social class. The program shows no preferences for demographic characteristics, which means that the program will evaluate your application solely on your academic qualifications. All admissions decisions will be made by the program, with no human input.

**A bank is deciding whether to give personal loans to applicants. The decision will be made in either of the following ways:**

A. By a loan officer who has preferences to approve loans of people who are financially eligible and also fit certain demographic categories such as race, gender, age or social class. It turns out that this loan officer’s preferences are for characteristics you do not possess, which makes it likely that the officer will be unfavorable towards you when evaluating your loan application

B. By a sophisticated computer program that has been programmed to automatically evaluate and approve loans for people who are financially eligible, irrespective of demographic factors such as nationality, gender age, or social class. The program shows no preferences for demographic characteristics, which means that the program will evaluate your loan application solely on your financial eligibility. All loan decisions will be made by the program, with no human input.

**A company is deciding who to hire for an open position. The decision will be made in either of the following ways:**

A. By a supervisor who has preferences to hire the most qualified candidates, who also fit certain demographic categories such as race, gender, age or social class. It turns out that this supervisor’s preferences are for characteristics you do not possess, which makes it likely that the supervisor will be unfavorable towards you when evaluating your application.

B. By a sophisticated computer program that has been programmed to automatically evaluate and identify the most qualified candidates, irrespective of demographic factors such as nationality, gender age, or social class. The program shows no preferences for demographic characteristics, which means that the program will evaluate your application solely based on your vocational qualifications. All hiring decisions will be made by the program, with no human input.

**A company is deciding which employees to layoff for a downsizing initiative that is required to keep the company in business. The decision will be made in either of the following ways:**

A.  By an HR manager who has preferences to retain the most productive people who also fit certain demographic categories such as race, gender, age or social class. It turns out that this manager's preferences are for characteristics you do not possess, which makes it likely that the manager will be unfavorable towards you when evaluating whether you should be laid off or not.

B.  By a sophisticated computer program that has been programmed to automatically identify and keep the most productive employees, irrespective of demographic factors such as nationality, gender age, or social class. The program shows no preferences for demographic characteristics, which means that the program will evaluate whether you should be laid off or not solely based on your productivity. All layoff decisions will be made by the program, with no human input.

**Uncertain Condition**

**A university is deciding which students to admit to their incoming class. The decision will be made in either of the following ways:**

A. By an admissions officer who has preferences to admit the best students who also fit certain demographic factors such as race, gender, age or social class. You do not know what characteristics matter to this admissions officer only that the officer could be either favorable or unfavorable towards you when evaluating your application

B.  By a sophisticated computer program that has been programmed to automatically identify and admit the best students, irrespective of demographic factors such as nationality, gender, age, or social class. The program shows no preferences for demographic characteristics, which means that the program will evaluate your application solely on your academic qualifications. All admissions decisions will be made by the program, with no human input.

**A bank is deciding whether to give personal loans to applicants. The decision will be made in either of the following ways:**

A.  By a loan officer who has preferences to approve loans of people who are financially eligible and also fit certain demographic categories such as race, gender, age or social class. You do not know what characteristics matter to this officer only that the officer could be either favorable or unfavorable towards you when evaluating your loan application.

B. By a sophisticated computer program that has been programed to automatically evaluate and approve loans for people who are financially eligible, irrespective of demographic factors such as nationality, gender age, or social class. The program shows no preferences for demographic characteristics, which means that the program will evaluate your loan application solely on your financial eligibility. All loan decisions will be made by the program, with no human input.

**A company is deciding who to hire for an open position. The decision will be made in either of the following ways:**

A. By a supervisor who has preferences to hire the most qualified candidates, who also fit certain demographic categories such as race, gender, age or social class. You do not know what characteristics matter to this supervisor only that the company could be either favorable or unfavorable towards you when evaluating your application.

B. By a sophisticated computer program that has been programmed to automatically evaluate and identify the most qualified candidates, irrespective of demographic factors such as nationality, gender age, or social class. The program shows no preferences for demographic characteristics, which means that the program will evaluate your application solely based on your vocational qualifications. All hiring decisions will be made by the program, with no human input.

**A company is deciding which employees to layoff for a downsizing initiative that is required to keep the company in business. The decision will be made in either of the following ways:**

A. By an HR manager who has preferences to retain the most productive people who also fit certain demographic categories such as race, gender, age or social class. You do not know what characteristics matter to this manager only that the manager could be either favorable or unfavorable towards you when evaluating whether you should be laid off or not.

B. By a sophisticated computer program that has been programmed to automatically identify and keep the most productive employees, irrespective of demographic factors such as nationality, gender age, or social class. The program shows no preferences for demographic characteristics, which means that the program will evaluate whether you should be laid off or not solely based on your productivity. All layoff decisions will be made by the program, with no human input.

S**TUDY 3**

You are a retail manager. You’ve been working at a large and very prestigious retail store in the US for most of your career and generally enjoy what you do. Your role is very well respected in your profession and generally people in your industry highly respect you and your work. As a result, many people in your industry have supported you in your career progression.

Last week you applied for a new managerial position at a large department store, where you and other applicants will have to perform an online job interview. Overall, when you interview for new roles you are very highly respected.

Please write down three potential upsides of having your current job, when applying for  a new position.

_______________________________

You are a retail manager. You’ve been working at a small and insignificant retail store in the US for most of your career and generally enjoy what you do. Your role is not very well respected in your profession and generally people in your industry look down upon you and your work. As a result, very few people in your industry have supported you in your career progression.

Last week you applied for a new managerial position at a large department store, where you and other applicants will have to perform an online job interview. Overall, when you interview for new roles you are looked down upon.

Please write down three potential upsides of having your current job, when applying for  a new position.

_______________________________
